# Patient and haemodynamic factors affecting intraoperative graft flow during coronary artery bypass grafting: an observational pilot study

**DOI:** 10.1038/s41598-020-69924-w

**Published:** 2020-07-31

**Authors:** Sang-Wook Lee, Jun-Young Jo, Wook-Jong Kim, Dae-Kee Choi, In-Cheol Choi

**Affiliations:** Department of Anesthesiology and Pain Medicine, Asan Medical Center, University of Ulsan College of Medicine, Seoul, Republic of Korea

**Keywords:** Cardiology, Medical research

## Abstract

Transit-time flow measurement (TTFM) is frequently used to evaluate intraoperative quality control during coronary artery bypass grafting (CABG) and has the ability to assess graft failure intraoperatively. However, perioperative factors affecting TTFM during CABG remain poorly understood. Patients who underwent CABG at a single institution between July 2016 and May 2018 were prospectively evaluated. TTFM and blood viscosity were measured haemodynamically, while mean flow (mL/min), pulsatility index, and diastolic filling were recorded. Arterial blood gas was analysed immediately after left internal mammary artery to left descending artery anastomosis and before sternal closure. Factors associated with TTFM were assessed using multiple linear regression analysis. We evaluated 57 of the 62 patients who underwent CABG during the study period, including 49 who underwent off-pump and 8 who underwent on-pump surgeries. Blood viscosity was not significantly associated with TTFM (*p* > 0.05). However, TTFM was significantly associated with body mass index, systolic blood pressure, and cardiac index (*p* < 0.05 each). In conclusion, maintaining the SBP in the perioperative period and maintaining the CI with inotropic support or fluid resuscitation can be important in improving blood flow of graft vessels after surgery.

## Introduction

Ischaemic heart disease (IHD) is a global leading cause of death according to the World Health Organization survey from 2000 to 2016^1^. The mortality rate of IHD is gradually decreasing in Western countries due to the development of diagnostic and therapeutic techniques for IHD, whereas it is gradually increasing in developing countries because of the rapid increase in westernised lifestyles^2^. Coronary artery bypass grafting (CABG) remains an important and a preferred treatment technique despite the development of various interventions for IHD. The early detection of postoperative graft failure after CABG is very important to prevent postoperative complications, such as refractory angina, myocardial infarction, arrhythmias, and even death^3^.

Transit-time flow measurement (TTFM), which utilises ultrasound to measure the flow velocity in blood vessels, is frequently used for the intraoperative assessment of graft quality in patients undergoing CABG^3–8^. TTFM is a non-invasive, easy-to-use method for real-time measurement of the graft flow velocity, does not require complex equipment, and provides numerical results^9,10^. Moreover, the graft flow velocity measured using this method is reported to be accurate and reproducible^11,12^.

Blood viscosity, an indicator of the stickiness of blood, can be measured as the degree of blood resistance to flow^13^. The most important factors influencing blood viscosity are haematocrit, red blood cell deformability, red blood cell aggregation, and plasma viscosity^14,15^, with haematocrit being the most important factor^14^. Plasma viscosity is a function of the water content and macromolecular components, including the types and concentrations of plasma proteins^14,16^. Blood viscosity varies with shear rate,therefore, blood is less viscous at high shear rates and more viscous at low shear rates, due to increased vessel diameter or a low flow rate^17^. Blood viscosity is expected to vary continuously during surgery because of intraoperative haemorrhage and subsequent fluid administration and transfusion.

The patency of a coronary artery bypass depends on several factors, including the nature of the coronary vessel, quality and type of grafts, collateral flow through the native coronary vessel, and construction of the anastomosis^18–20^. Slower TTFM during CABG has been reported to be associated with a greater likelihood of graft failure after surgery^21^. Therefore, the velocity of blood flow through the graft vessels measured during surgery can affect patient prognosis and is, thus, necessary to determine factors affecting blood flow velocity, especially those than can be controlled^3,22–24^. However, to our knowledge, factors affecting the blood flow velocity of graft vessels, including those affecting changes in viscosity during surgery, have not yet been determined. Therefore, this study aimed to investigate the effect of blood viscosity on the velocity of blood flow through graft vessels measured during surgery in patients undergoing CABG.

## Methods

### Study population

The protocol of this observational study was approved by the Institutional Review Board of Asan Medical Center, and the study was registered at the Korean Clinical Trials Registry (KCT0002047) and followed the guidelines of the Helsinki Declaration. Written informed consent was obtained prospectively from each patient. Patients were prospectively included if they underwent on-pump CABG or off-pump coronary artery bypass (OPCAB) at a single institution between July 2016 and May 2018. Patients were excluded if they underwent an emergency surgery; had poor left ventricular systolic function, defined as preoperative ejection fraction < 40% on preoperative echocardiogram; had not undergone left internal mammary artery (LIMA) to left anterior descending artery (LAD) anastomosis; had preoperative arrhythmias such as atrial fibrillation; or refused participation. All clinical data were obtained from the electronic medical records system of our institution.

### Anaesthesia and perioperative management

After the administration of 0.1 mg/kg midazolam, loss of consciousness was observed, followed by the administration of 0.8 mg/kg rocuronium to induce general anaesthesia. Subsequently, propofol and remifentanil were continuously injected using a target controlled infusion pump (ORCHESTRA BASE PRIMEA; Fresenius Kabi, Brezins, France) to maintain general anaesthesia^25^. All patients received volume-controlled mechanical ventilation with a tidal volume of 8 mL/kg of the ideal body weight, with 50% inspired oxygen during surgery without positive end expiratory pressure^25^. Although cardiac preload was maintained with crystalloids or colloids, patients who showed reductions in the mean arterial pressure and/or cardiac index (CI) during surgery were administered inotropic agents such as dobutamine or norepinephrine and a vasopressor such as phenylephrine^25^. When haemoglobin concentration dropped < 8 g/dL, the transfusion of packed red blood cells was considered^25^. A cell salvage device (AUTOLOG, Medtronic Inc., Minneapolis, MN) was used in all patients who participated in the study to reinfuse salvaged blood before the end of surgery^25^. Throughout the surgery, all patients were continuously administered isosorbide dinitrate and a calcium channel blocker such as diltiazem.

All surgical procedures were performed by five cardiac surgeons highly experienced in on-pump CABG or OPCAB. All patients underwent median sternotomy. Minimal invasive surgery was excluded from this study for consistency of surgical procedures.

All patients were transferred to the intensive care unit (ICU) after surgery and then moved back to the general ward when clinical signs were stable or the patients no longer needed ICU monitoring and care^25^.

### Clinical variables and perioperative variables

Demographic variables recorded included patient age, sex, weight, height, and body mass index (BMI), and preoperative variables included previous medical history such as medications, EuroSCORE (II, logistic), and ejection fraction of the left ventricle on preoperative echocardiography. Laboratory data included haemoglobin concentration; haematocrit; and serum concentrations of blood urea nitrogen, creatinine, albumin, cholesterol, triglycerides, high-density lipids, low-density lipids, creatine kinase-MB, and Troponin I. Intraoperative data included anaesthesia time; surgery time; size of the graft vessel; intraoperative total fluid volume including transfusions; and haemodynamic parameters, including heart rate, mean arterial blood pressure, pulse oximetry, central venous pressure, cerebral oximetry, pulmonary artery pressure, CI, systemic vascular resistance index, and arterial blood gas analysis. Postoperative variables included the duration of mechanical ventilation, length of ICU stay, length of hospital stay, and postoperative complications. Postoperative complications included myocardial infarction, atrial fibrillation, wound complication, acute kidney injury, and death.

### Transit-time flow measurement

The primary outcome of this study was the mean flow rate (MFR) of TTFM, an ultrasound measure of the velocity of blood flow through the blood vessel. TTFM is based on the measured difference in time required for blood flow between two ultrasonic signals emitted by a probe^4,26,27^. The TTFM consists of three components: MFR, pulsatility index (PI), and diastolic filling (DF). The MFR of TTFM is expressed as millilitres per minute (mL/min)^28^. TTFM in this study was measured twice, immediately after LIMA to LAD anastomosis and before sternal closure, using a VERI-Q Flowmeter (Medistim ASA, Oslo, Norway). MFR, PI, and DF were recorded whenever TTFM was measured. In this study, only TTFM measured in LIMA to LAD anastomosis was used for the analysis to minimise the effect of various types of graft vessels on the measurements. For other graft vessels harvested from different sites such as the saphenous vein or radial artery, the measurements may be affected by the graft vessels’ condition and anatomical variation and have to be made in similar clinical settings in all patients. Therefore, LIMA to LAD was selected as the measurement site in this study.

### Viscosity of graft flow measurements

Blood viscosity, measured as systolic and diastolic blood viscosity, is dependent on blood pressure, which changes with every cardiac cycle^29–31^. Systolic blood viscosity is dependent on haematocrit and plasma viscosity and is highly affected by the volume of intraoperative fluid infusion^29,30^. In contrast, diastolic blood viscosity is affected by many factors, including platelet counts and concentrations of immune complexes, triglycerides, and cholesterol^29,30^. TTFM and blood viscosity were measured at the same time. Immediately after measuring the velocity of blood flow through the graft vessel, 3 mL blood was collected in an EDTA container and refrigerated at 4 °C, while systolic and diastolic blood viscosity were measured using HEMOVISTER (Pharmode Inc., Seoul, Korea).

### Sample size and clinical data

The absence of previous studies or data from pilot studies prevented the calculation of the correct sample size. Assuming a first type error (α) of 0.05 and a second type error (β) of 20%, the power would be 80% when the expected effect size (ƒ) was set at 0.15 and the prediction factor at 5. Thus, 91 subjects were regarded as adequate, and assuming a 10% dropout rate, a total of 100 subjects was estimated as sufficient.

### Statistical analysis

Categorical variables are expressed as numbers and percentages, and continuous variables as means and standard deviations. Categorical variables were compared using the Pearson *χ*^2^ test or Fisher’s exact test, whereas continuous variables were compared using Student’s *t* test or the Mann–Whitney *U*-test. To investigate the associations between TTFM and clinical variables, including blood viscosity while accounting for the clustering effect of the repeated measurements within each patient, we used linear mixed model with random intercept for patients. TTFM was included as an outcome variable after log-transformation to achieve normality. Variables with *p* < 0.1 on univariate models and clinically meaningful variables were entered into the multivariable linear mixed model, with backward elimination procedures used to determine the independent variables associated with TTFM. The result of the final model was expressed as point estimate and 95% confidence intervals (CIs) of β coefficients. All measured variables at both measurement times were included in the analysis because of the small cohort size. All statistical analyses were performed using “R” statistical software (R ver. 3.5.1.), with *p* values < 0.05 considered statistically significant.

## Results

**Figure 1:**
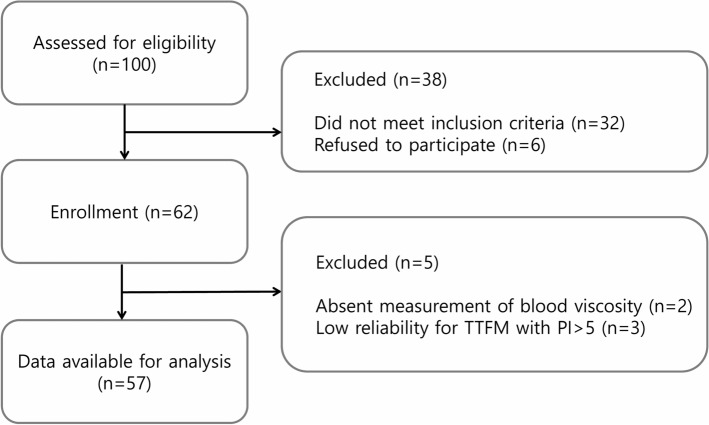
Flow diagram of the study. *TTFM* transit-time flow measurement, *PI* pulsatility index.

Of the 100 consecutive patients who underwent CABG between July 2016 and May 2018, 38 who met the exclusion criteria and two other patients in whom blood viscosity was not measured were excluded. Considering that blood flow < 20 mL/min combined with PI > 5 indicates technically inadequate grafts^32^, three additional patients with PI > 5 were also excluded (Fig. 1). Thus, this study finally included 57 patients (45 men and 12 women), with a mean age of 64.3 ± 8.3 years. Of these 57 patients, 49 (86.0%) underwent off-pump and 8 (14.0%) underwent on-pump CABG. Baseline demographic and clinical characteristics of the study patients are shown in Table 1.

**Table 1:** Baseline clinical characteristics and perioperative data of the enrolled patients.

Variable	Number (percentage) or mean ± SD	Variable	Number (percentage) or mean ± SD
Number of patients	57	LDL (mg/dL)	90.3 ± 35.7
Off-pump surgery	49 (86.0%)	CK-MB (ng/mL)	1.4 ± 1.1
Demographics		Troponin I (ng/dL)	0.7 ± 2.1
Age (year)	64.3 ± 8.3	Intraoperative data	
Sex (male)	45 (79.0%)	Anaesthetic time (min)	285.33 ± 46.53
Height (cm)	163.1 ± 7.6	Surgery time (min)	226.58 ± 50.10
Weight (kg)	66.0 ± 10.6	Size of the graft vessel (mm)	1.56 ± 0.25
BMI (kg/m^2^)	24.7 ± 3.4	Intraoperative total fluid (mL)	3,647.40 ± 1,457.06
Medical history		Crystalloid (mL)	2,600.00 ± 1,067.88
HTN	39 (68.4%)	Colloid (mL)	583.33 ± 314.72
DM	27 (47.4%)	Packed RBC (mL)	207.89 ± 357.91
CRF	8 (14.0%)	Urine output (mL)	494.18 ± 462.21
History of MI	4 (7.0%)	MFR of TTFM (mL/min)	23.20 ± 13.53
Status of post PCI	11 (19.3%)	PI of TTFM	2.19 ± 0.78
Statin medication	39 (68.4%)	DF of TTFM (%)	72.37 ± 10.24
EuroSCOREII	1.7 ± 1.3	Viscosity S (mPa.s)	3.08 ± 0.43
EuriSCORE (logistic)	4.0 ± 3.6	Viscosity D (mPa.s)	8.09 ± 2.46
Left ventricular EF (%)	58.8 ± 7.9	Postoperative data	
Laboratory data		ICU stay (hours)	39.9 ± 24.7
Hb (g/dL)	12.7 ± 1.6	Hospital stay (days)	8.7 ± 4.6
Hct (%)	38.0 ± 4.5	MV time (hours)	9.8 ± 9.6
BUN (mg/dL)	18.5 ± 9.4	Postoperative complications	9 (15.8%)
Creatinine (mg/dL)	1.4 ± 1.5	Myocardial infarction	0 (0% )
Albumin (g/dL)	3.7 ± 0.6	Atrial fibrillation	6 (10.5%)
Total cholesterol (mg/dL)	142.9 ± 37.2	Wound complication	1 (1.8%)
Triglyceride (mg/dL)	169.0 ± 141.2	Acute kidney injury	2 (3.5%)
HDL (mg/dL)	41.7 ± 12.0	Death	0 (0% )

*SD* standard deviation, *BMI* body mass index, *HTN* hypertension, *DM* diabetes mellitus, *CRF* chronic renal failure, *MI* myocardial infarction, *PCI* percutaneous coronary intervention, *Hb* haemoglobin, *Hct* haematocrit, *BUN* blood urea nitrogen, *HDL* high-density lipoprotein, *LDL* low-density lipoprotein, *CK-MB* creatine kinase-muscle/brain, *EF* ejection fraction, *RBC* red blood cell, *MFR* mean flow rate, *TTFM* transit-time flow measurement measured at the anastomosis from LIMA to LAD, *PI* pulsatility index, *DF* diastolic filling, *Viscosity S* systolic blood viscosity, *Viscosity D* diastolic blood viscosity, *ICU* intensive care unit, *MV* mechanical ventilation.

**Figure 2:**
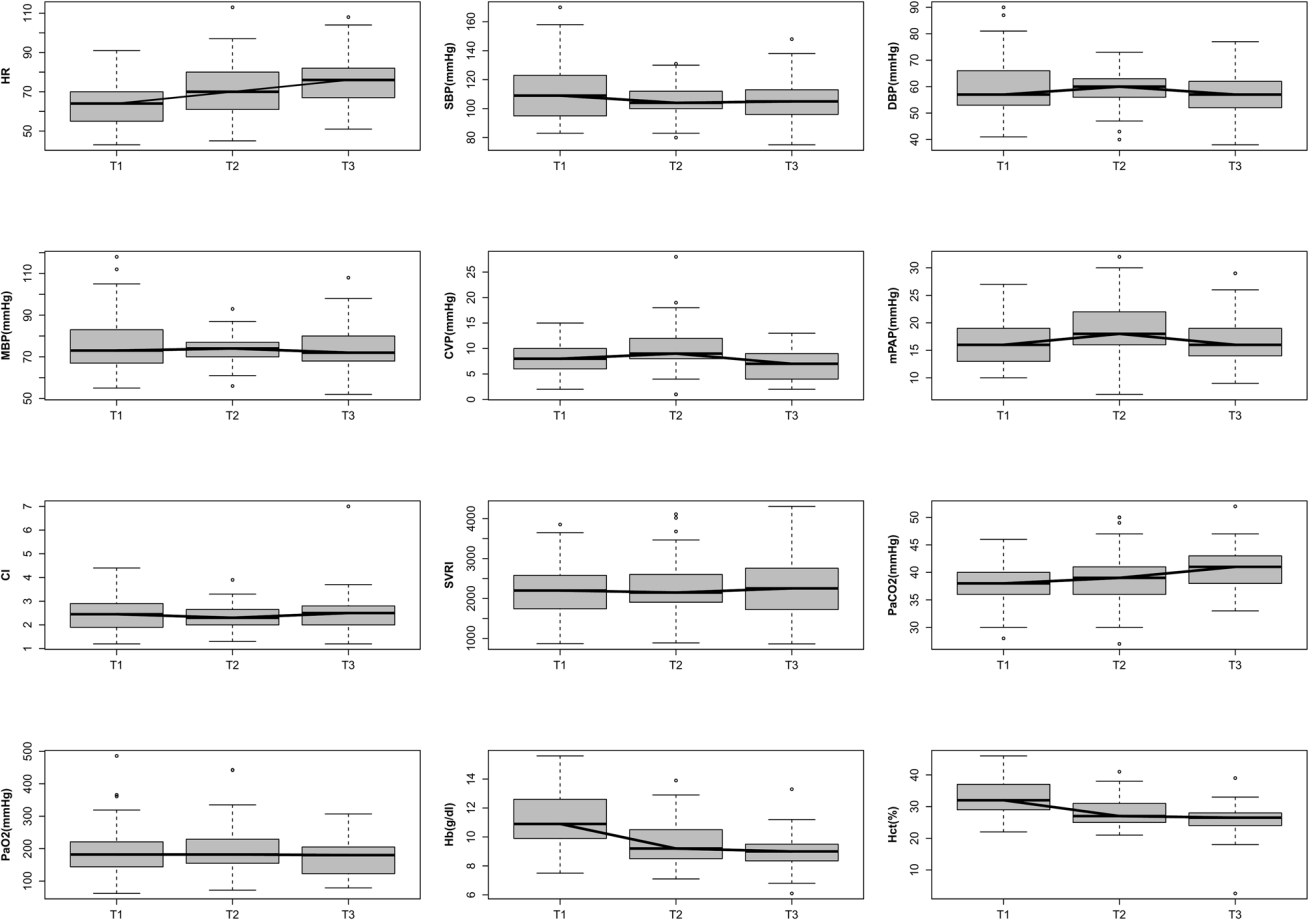
Intraoperative and haemodynamic findings. T1, after anaesthesia induction. T2, after left internal mammary artery (LIMA) to left anterior descending artery (LAD) anastomosis. T3, before sternal closure. *HR* heart rate, *SBP* systolic blood pressure, *DBP* diastolic blood pressure, *MBP* mean blood pressure, *CVP* central venous pressure, *mPAP* mean pulmonary artery pressure, *CI* cardiac index, *SVRI* systemic vascular resistance index, *PaCO*_*2*_ partial pressure of carbon dioxide, *PaO*_*2*_ partial pressure of oxygen, *Hb* haemoglobin, *Hct* haematocrit.

Intraoperative data are shown in Table 1 and haemodynamic findings in Fig. 2. Mean blood pressure, diastolic blood pressure, central venous pressure, and mean pulmonary arterial pressure increased after LIMA to left descending artery (LAD) anastomosis rather than immediately after induction but later decreased before sternal closure (Fig. 2). Conversely, systolic blood pressure and the CI decreased after anastomosis rather than immediately after induction but increased before sternal closure. Table 2 shows the graft vessel type and TTFM parameters measured in each graft vessel in patients enrolled in this study. The average ICU stay was 40 h, average hospital stay was approximately 9 days, and mean mechanical ventilation time was approximately 10 h. Nine patients experienced postoperative complications, including six with atrial fibrillation, one with wound complications, and two with acute kidney injury. Postoperative outcomes are summarised in Table 1.

**Table 2:** The values of the TTFM parameters for each anastomosis site.

Anastomosis site	Number of cases	Mean flow rate	Pulsatility index	Diastolic filling
LIMA to LAD	57	23.20 ± 13.53	2.19 ± 0.78	72.37 ± 10.24
SVG to OM	50	38.70 ± 32.85	3.35 ± 2.42	54.09 ± 23.55
PDA	35	28.38 ± 22.27	2.59 ± 1.87	53.21 ± 23.33
DI	8	33.33 ± 31.30	2.62 ± 0.90	64.00 ± 6.90
PL	1	42.00	2.4	78.00
RI	6	27.50 ± 16.26	3.45 ± 0.92	63.50 ± 19.09
dRCA	6	34.60 ± 19.44	2.08 ± 1.03	44.80 ± 27.18

*LIMA* left internal mammary artery, *LAD* left anterior descending artery, *OM* obtuse marginal branch artery, *PDA* posterior descending artery, *DI* diagonal artery, *PL* posterolateral artery, *RI* ramus intermedius artery, *dRCA* distal right coronary artery.

**Figure 3:**
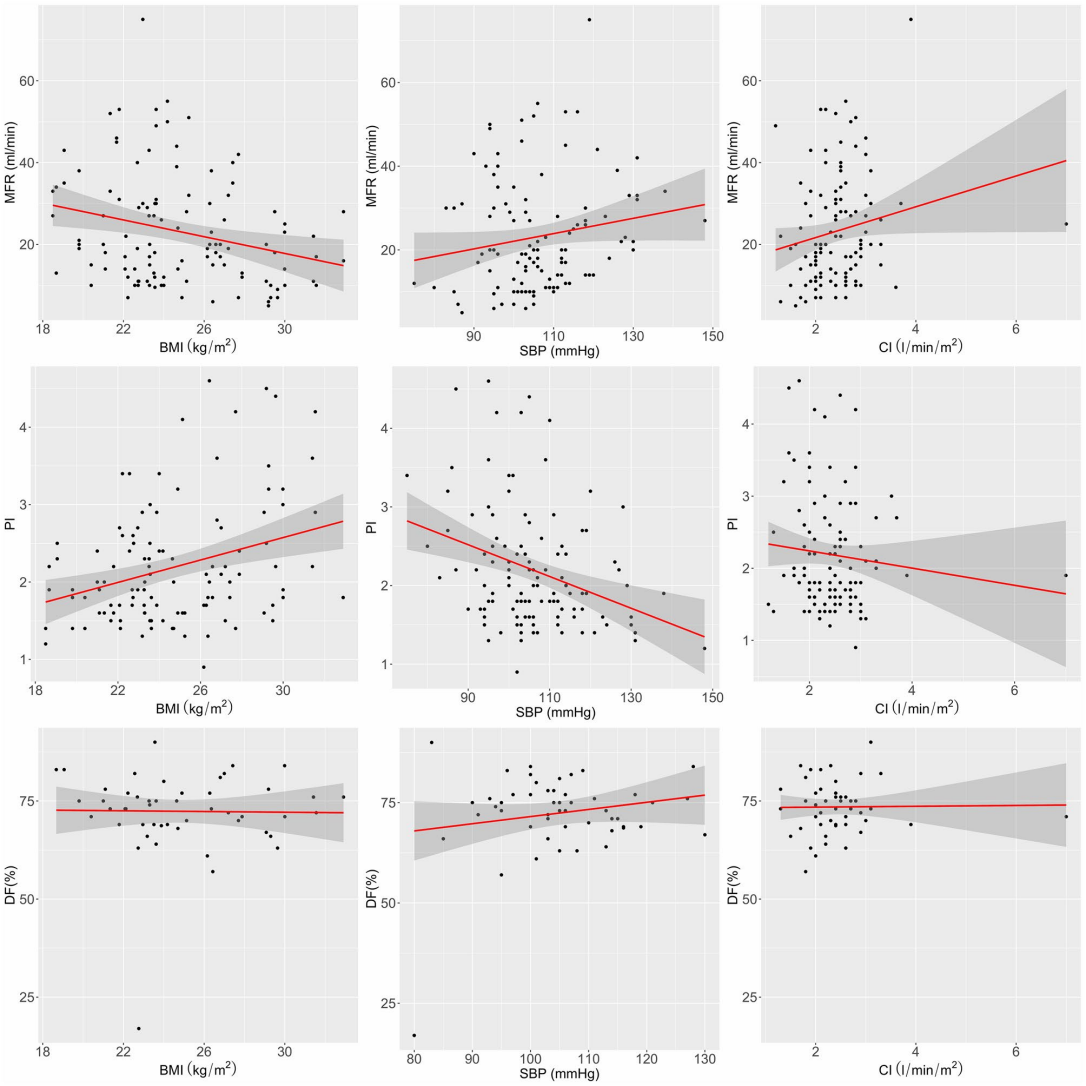
Scatter plot showing the correlation between the three parameters of TTFM and BMI, SBP, and CI. *MFR* mean flow rate, *PI* pulsatility index, *DF* diastolic filling, *TTFM* transit-time flow measurement, *BMI* body mass index, *SBP* systolic blood pressure, *CI* cardiac index.

**Figure 4:**
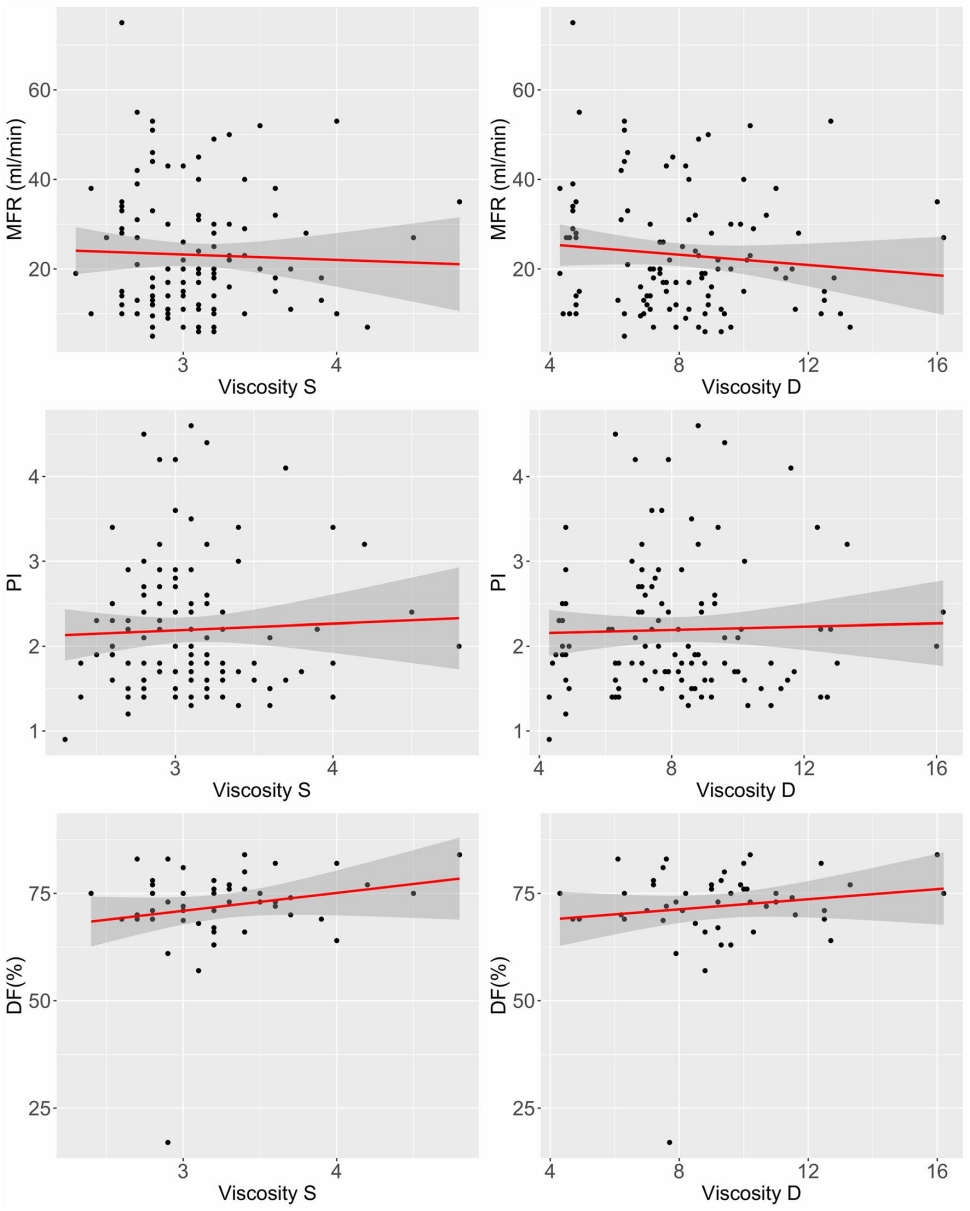
Scatter plot showing the correlation between the three parameters of TTFM and viscosity. *MFR* mean flow rate, *PI* pulsatility index, *DF* diastolic filling, *TTFM* transit-time flow measurement. Viscosity S, systolic blood viscosity; Viscosity D, diastolic blood viscosity.

Table 3 shows the results of univariate linear regression analysis regarding the effect of perioperative factors on the MFR, PI, and DF of TTFM. The calculation of p-value in this analysis was often in error because of many missing values in the DF of TTFM. The results of univariate and multivariate linear regression analysis of factors affecting the MFR of TTFM are summarised in Table 4. Univariate linear regression showed that female sex, BMI, ejection fraction on preoperative echocardiogram, volumes of total fluid and packed red blood cells infused intraoperatively, heart rate, systolic blood pressure, mean blood pressure, oxygen saturation on pulse oximetry, and CI were significantly associated with the MFR of TTFM (*p* < 0.1 each) (Table 4). These variables were used to construct a multiple linear regression model, which found that BMI (*p* = 0.008), systolic blood pressure (*p* = 0.023), and CI (*p* = 0.039) were independently associated with the MFR of TFFM (Fig. 3). The volume of total fluid infused intraoperatively tended to be significantly associated with the MFR of TFFM (*p* = 0.081). However, the MFR of TTFM was not associated with blood viscosity (Fig. 4).

**Table 3:** The *p *value for the effect of perioperative factors on several parameters of TTFM at the graft from LIMA to LAD.

	TTFM		
	MFR	PI	DF
Age	0.796	0.949	0.095
Sex (female)	0.060	0.335	0.329
BMI	0.014*	0.001**	0.883
HTN	0.137	0.910	0.870
DM	0.623	0.270	0.017*
S/P PCI	0.147	0.002**	0.060
StatinMx	0.152	0.007**	0.165
EuroSCORE II	0.769	0.414	0.863
EuroSCORE L	0.758	0.983	0.728
preHb	0.116	0.289	0.172
preAlb	0.391	0.139	0.718
preEF	0.038*	0.933	0.789
CABG	0.467	0.252	0.039*
LADsize	0.410	0.799	0.656
tFluid	0.045*	0.800	0.548
pRBC	0.008*	0.182	0.414
HR	0.058	0.073	0.371
SBP	0.003**	0.0002***	0.224
DBP	0.259	0.0054**	0.483
MBP	0.006**	0.0001***	0.465
SpO_2_	0.008**	0.0009***	0.154
CVP	0.132	0.921	0.116
CI	0.022*	0.264	0.614
SVRI	0.646	0.733	0.456
PaCO_2_	0.783	0.723	0.333
PaO_2_	0.279	0.102	0.196
Viscosity S	0.593	0.491	0.163
Viscosity D	0.414	0.616	0.292

*TTFM* transit-time flow measurement, *MFR* mean flow rate measured, *PI* pulsatility index, *DF* diastolic filling, *BMI* body mass index, *HTN* hypertension, *DM* diabetes mellitus, *S/P PCI* status of post-percutaneous coronary intervention, *Statin Mx* history of statin medication, *EuroSCORE L* logistic EuroSCORE, *preHb* preoperative haemoglobin, *preAlb* preoperative albumin, *preEF* preoperative ejection fraction, *CABG* coronary artery bypass graft, *LAD* left anterior descending artery, *tFluid* total fluid administered intraoperatively, *pRBC* packed red blood cells administered intraoperatively, *HR* heart rate, *SBP* systolic blood pressure, *DBP* diastolic blood pressure, *MBP* mean blood pressure, *SpO*_*2*_ oxygen saturation, *CVP* central venous pressure, *CI* cardiac index, *SVRI* systemic vascular resistance index, *PaCO*_*2*_ partial pressure of carbon dioxide, *PaO*_*2*_ partial pressure of oxygen, *Viscosity S* systolic blood viscosity, *Viscosity D* diastolic blood viscosity.

*p < 0.05; **p < 0.01; ***p < 0.001.

**Table 4:** Univariate and multivariate linear regression analysis of factors associated with the MFR of TTFM.

	Univariate				Multivariable			
	Beta estimate	95% CIs		*p* value	Beta estimate	95% CIs		*p* value
		Lower	Upper			Lower	Upper	
Age	− 0.002	− 0.019	0.015	0.796				
Sex (female)	− 0.310	− 0.634	0.013	0.060				
BMI	− 0.048	− 0.086	− 0.010	0.014	− 0.050	− 0.085	− 0.014	0.008
HTN	0.216	− 0.071	0.503	0.137				
DM	0.067	− 0.205	0.339	0.623				
S/P PCI	− 0.249	− 0.587	0.090	0.147				
StatinMx	0.209	− 0.079	0.496	0.152				
EuroSCORE II	0.015	− 0.088	0.119	0.769				
EuroSCORE L	0.006	− 0.033	0.045	0.758				
preHb	0.067	− 0.017	0.152	0.116				
preAlb	0.094	− 0.124	0.312	0.391				
preEF	− 0.018	− 0.035	− 0.001	0.038				
CABG	0.143	− 0.248	0.533	0.467				
LADsize	0.225	− 0.318	0.768	0.410				
tFluid	0.398	0.009	0.787	0.045	0.326	− 0.034	0.685	0.081
pRBC	0.001	0.000	0.001	0.008				
HR	0.008	0.000	0.017	0.058				
SBP	0.013	0.005	0.021	0.003	0.009	0.002	0.017	0.023
DBP	0.008	− 0.006	0.023	0.259				
MBP	0.017	0.005	0.028	0.006				
SpO_2_	0.796	0.213	1.378	0.008				
CVP	− 0.018	− 0.041	0.006	0.132				
CI	0.183	0.028	0.339	0.022	0.158	0.012	0.305	0.039
SVRI	0.000	0.000	0.000	0.646				
PaCO_2_	− 0.003	− 0.028	0.021	0.783				
PaO_2_	0.001	− 0.001	0.003	0.279				
ViscosityS	− 0.061	− 0.287	0.166	0.593				
ViscosityD	− 0.016	− 0.054	0.023	0.414				

*TTFM* transit-time flow measurement, *MFR* mean flow rate, *CIs* confidence intervals, *BMI* body mass index, *HTN* hypertension, *DM* diabetes mellitus, *S/P PCI* status of post-percutaneous coronary intervention, *Statin Mx* history of statin medication, *EuroSCORE L* logistic EuroSCORE, *preHb* preoperative haemoglobin, *preAlb* preoperative albumin, *preEF* preoperative ejection fraction, *CABG* coronary artery bypass graft, *LAD* left anterior descending artery, *tFluid* total fluid administered intraoperatively, *pRBC* packed red blood cells administered intraoperatively, *HR* heart rate, *SBP* systolic blood pressure, *DBP* diastolic blood pressure, *MBP* mean blood pressure, *SpO*_*2*_ oxygen saturation, *CVP* central venous pressure, *CI* cardiac index, *SVRI* systemic vascular resistance index, *PaCO*_*2*_ partial pressure of carbon dioxide, *PaO*_*2*_ partial pressure of oxygen, *Viscosity S* systolic blood viscosity, *Viscosity D* diastolic blood viscosity.

## Discussion

TTFM has been reported to be important for determining the success of grafts during surgery^22^ and predicting the risk of graft failure after surgery^21^, making it the most widely used method of intraoperative graft quality control in patients undergoing CABG^3–8^. However, no prior study investigating perioperative factors affecting TTFM during CABG has been conducted,therefore, the present study is one of the first to assess perioperative factors affecting flow measurements in graft vessels.

Although this study was designed to determine the association between blood viscosity and TTFM, no such significant association was observed. Because blood is a non-Newtonian fluid, its viscosity is affected by shear rate^17^. Blood viscosity is higher at low shear rates and lower at high shear rates^17^. Coronary artery blood flow is not at steady-state, making its viscosity inconstant. Furthermore, previous studies have reported a significant increase in blood viscosity during and after cardiac surgery^33^. Cardiac surgery can cause abnormal blood rheological characteristics that may be associated with lung dysfunction and endothelial damage^34^. In addition, blood viscosity changes due to systemic inflammatory and thrombotic responses during cardiac surgery^33^. Although blood viscosity was measured twice during surgery and was divided into systolic and diastolic viscosity in this study, it changes continuously according to the blood flow during surgery, making it difficult to determine the real-time relationship between viscosity and TTFM. Despite the limitations of the measuring methods, blood viscosity plays an important role in haemodynamics, thrombosis, and inflammation and also affects the diagnosis and treatment of cardiovascular diseases^35–40^. Because we were unable to determine the effect of blood viscosity on postoperative clinical outcomes, including postoperative graft failure, further studies are needed.

Interestingly, we found that BMI was significantly associated with the MFR of TTFM, which is in agreement with findings showing that high BMI or obesity is associated with coronary endothelial dysfunction, an early stage of coronary atherosclerosis that may involve the epicardial and/or resistance vessels^41,42^. The negative effects of obesity on coronary circulation include immediate changes in coronary arterial vasomotor responsiveness and the development of occlusive coronary artery disease^43,44^, as well as the generation of adipocyte-derived adipokines^45,46^. Several diseases in obese patients caused by coronary microvascular inflammation have been associated with cellular mechanisms that control the secretion of adipokines and proinflammatory cytokines from adipose tissue^47^. This association between BMI and TTFM may indicate that metabolic dysfunction associated with obesity also affects coronary blood flow.

Factors significantly associated with the MFR of TTFM in the present study included systolic blood pressure and CI, suggesting that the haemodynamic status is an important determinant of TTFM. However, in this study, the contribution or effect of each factor could not be determined.

We expected that diastolic blood pressure would more likely affect the MFR of TTFM than systolic blood pressure because coronary perfusion pressure is determined by the difference between aortic diastolic pressure and left ventricular end-diastolic pressure (LVEDP)^48^. Surprisingly, we found that systolic blood pressure was associated with the MFR of TTFM, whereas diastolic blood pressure was not. Blood is supposed to flow from areas of high blood pressure to areas of low blood pressure^49^. Therefore, blood flow increases in proportion to the difference in blood pressure between the two blood vessels^49^. In this study, the measured blood flow is the value obtained by placing the probe at the position close to the anastomosis site in the graft vessel, after connecting the graft vessel to the native coronary vessel. Because coronary blood flow is determined by the coronary perfusion pressure^50^^,^ which is the difference between the diastolic pressure and left ventricle end-diastolic pressure, native coronary blood flow is primarily determined by the diastolic pressure. We believe that the blood flow from LIMA to LAD will be mainly affected by the difference between systolic and diastolic blood pressures. Therefore, systolic pressure may have a greater effect on the blood flow of the graft vessel than diastolic pressure according to the results of this study. As expected, CI was associated with the MFR of TTFM because cardiac output is an important determinant of both coronary blood flow and blood flow through the engrafted vessel.

This study has several limitations. The small sample size is one of the main limitations of this study. We originally intended to enrol 91 patients in this study, but the number of patients who were finally enrolled decreased significantly because more patients were excluded from the study than expected. Nevertheless, our findings represent an important first step in evaluating perioperative factors affecting the measurement of blood flow through graft vessels. Studies investigating factors affecting TTFM involving a large sample size are needed to determine the extent of their effects on TTFM. TTFM and viscosity are continuous variables that change from time to time. Therefore, it is desirable to look at these changes according to the various measurement points. However, in actual clinical situations other than laboratory studies, it is difficult to take measurements at multiple time points, and the variables need to be measured at two important time points according to our judgement. Therefore, although the measurements were not sufficient, the study design considered the clinical situation by collecting the minimum data necessary for analysis. Additionally, there was an important coronary anatomical variation among patients in this study, and the types of graft blood vessels also varied, making it difficult to classify and analyse them. Therefore, we believe that only the measurement from LIMA to LAD, which is the single type of graft blood vessels was used in the analysis to prevent the unreliability of the results by using various types of graft blood vessels Because this was not a laboratory study, it was difficult to control certain variables due to the interference of other external factors; however, the research was designed taking this into account, considering that the research was conducted in a clinical setting. In future studies, it is thought that better results can be obtained if values from measurements at multiple time points are used and also by performing subgroup analyses according to the quality of the patient's inflow conduit and outflow target. Another limitation of this study was that surgeries were performed by five different surgeons, which may have affected the TTFM. This affects the results of the surgery, but measurement errors may occur as the person measuring the TTFM changes. In future research, it is suggested that this limitation be considered.

## Conclusion

This study found no significant relationship between blood viscosity and intraoperative graft flow. In contrast, BMI, systolic blood pressure, and CI were significantly associated with blood flow through graft vessels. The main implication of this study was that although it is acutely impossible to change the BMI, maintaining the SBP, which is to be kept higher if necessary in the perioperative period and maintaining the CI, that is to be optimised with inotropic support or fluid resuscitation can play an important role in improving blood flow of graft vessels after surgery. Additional studies are needed to determine the extent to which individual factors affect TTFM and how factors related to TTFM affect clinical outcomes.

## Data Availability

All data regarding this study is available upon reasonable request to the corresponding author.
